# Aberrant Peripheral Immune Function in a Good Syndrome Patient

**DOI:** 10.1155/2018/6212410

**Published:** 2018-04-23

**Authors:** Xian Chen, Jie-xin Zhang, Wen-wen Shang, Wei-ping Xie, Shu-xian Jin, Fang Wang

**Affiliations:** ^1^Department of Laboratory Medicine, The First Affiliated Hospital of Nanjing Medical University, Nanjing 210029, China; ^2^National Key Clinical Department of Laboratory Medicine, Nanjing 210029, China; ^3^Department of Respiratory and Critical Care Medicine, The First Affiliated Hospital of Nanjing Medical University, Nanjing 210029, China

## Abstract

Good's syndrome (GS) is often accompanied by recurrent respiratory infections and chronic diarrhea. The main purpose was to evaluate the peripheral immune status of a GS patient after thymoma resection. Twenty healthy volunteers were recruited as healthy controls (HCs). Flow cytometry was applied to determine the proportions of circuiting CD4^+^ T cells, CD8^+^ T cells, *γδ*T cells, and regulatory T (Treg) cells in our GS patient. We also examined the proliferation capability of ex vivo CD4^+^ T cells and detected the levels of cytokines interferon- (IFN-) *γ* and interleukin-17A secreted by ex vivo immune cells from this GS patient. Compared with healthy control subjects, this GS patient had fewer B cells, an inverted ratio of CD4^+^/CD8^+^ cells, and more Treg cells in his peripheral blood. Additionally, the patient's V*δ*2 T cell levels were significantly decreased despite having a normal percentage of *γδ*T cells. Ex vivo peripheral CD4^+^ T cells from the patient showed insufficient proliferation and division potential as well as excessive expression of PD-1. Moreover, IFN-*γ* was predominantly derived from CD8^+^ T cells in this GS patient, rather than from CD4^+^ T cells and *γδ*T cells. This GS patient had impaired T and B cell immunological alternations and cytokine disruptions after thymectomy. Detailed research should focus on therapies that can adjust the immune status in such patients for a better outcome.

## 1. Introduction

Thymoma is an epithelial tumor originating from the thymus, and it is linked to various immunological disorders according to different subtypes. GS, a subtype of thymoma firstly described in 1954 by Dr. Good [1], was a rare disease accompanied by respiratory system abnormality with recurrent bronchitis, sinusitis, pneumonia, and chronic diarrhea. To date, patients diagnosed with GS mainly distributed in Europe and America and totally 47 cases emerged in China [2]. GS is typically detected based on a diagnosis of thymoma in combination with characteristic clinical laboratory test results, such as a low number or even absence of peripheral B cells, hypogammaglobulinemia, CD4^+^ T lymphopenia, and an inverted ratio of CD4^+^/CD8^+^ cells. This condition may also have autoimmune manifestations, such as myasthenia gravis or diabetes mellitus [3]. Thymectomy is an optimal treatment strategy that hinders locally aggressive growth and metastasis of the thymoma, and this procedure is widely applied clinically in conjunction with regular immunoglobulin infusions to restore immune balance [4].

In GS patients, an imbalance of immune cells and cytokines usually leads to tumorigenesis and recurrent chronic infections. Regulatory T (Treg) cells maintain self-tolerance and control autoimmunity, but they can also contribute to immunosuppression by restraining effective anti-infection immunity [5]. Interferon-gamma (IFN-*γ*) is an essential cytokine responsible for the cytotoxic effects exerted by T helper type 1 cells, and interleukin- (IL-) 17A, a proinflammatory cytokine, actively participates in chronic infection through the IL-17 and IL-23 signaling pathways. However, to date, little is known about the role of peripheral Treg cells or the involvement of these two cytokines in GS patients. Here, we detected circulating immune cells, including Treg cells, and inflammatory cytokines to assess the peripheral immunological status of a GS patient. Our findings may provide the foundation for new treatment strategies for recurrent postthymectomy respiratory infections in GS patients.

## 2. Materials and Methods

### 2.1. Patient and Specimens

This study was approved by the Ethical Committee of the First Affiliated Hospital of Nanjing Medical University (Nanjing, China). All presurgery information of the GS patient was collected from previous medical histories. Age-matched donors with no family history of autoimmune disease or tumors were considered as HCs.

### 2.2. Flow Cytometry Analysis

Peripheral blood samples were collected in morning fasting status. The fluorochrome-conjugated monoclonal antibodies used in this study were as follows: FITC-conjugated anti-CD4, FITC-conjugated anti-CD8, APC-conjugated anti-CD25, APC-conjugated anti-CD28, BV421-conjugated anti-Foxp3, PE-conjugated anti-CD39, BV421-conjugated anti-TCR*γδ*, PE-conjugated anti-V*δ*2, APC-conjugated anti-IL-17A, PE-Cy7-conjugated anti-IFN-*γ* (Biolegend, San Jose, CA, USA), PE-conjugated anti-PD-1 and PE-conjugated anti-PD-L1(BD Bioscience, San Jose, CA, USA), FITC-conjugated anti-V*δ*1 (Abcam, USA), and relevant isotype controls. For extracellular staining, appropriate mAbs of surfacemarkers were added to samples in the dark at room temperature for 20 minutes. For intracellular staining, peripheral blood mononuclear cells (PBMCs) were stimulated with PMA (500 ng/mL), ionomycin (1 *μ*g/mL), and BFA (2 *μ*g/mL) at 37°C for 5 hours. For intranuclear staining, PBMCs were incubated with fixation/permeabilization buffer for 30 minutes at 4°C. Anti-Foxp3 antibody was added to the cell pellet for 30 minutes in the dark at 4°C. Labeled cells were then measured by a flow cytometer (BD Arial II; BD Bioscience, San Jose, CA, USA), and the data were analyzed via FlowJo 6.0 software (Tree Star).

### 2.3. Proliferation Assay

We performed proliferation assay in round-bottomed 96-well plates. PBMCs (1 × 10^6^) isolated from the GS patient and healthy donors were labeled with CFSE (0.5 *μ*M; Invitrogen) and incubated with anti-CD3 (2 *μ*g/mL) and anti-CD28 (2 *μ*g/mL) (eBioscience, San Diego, CA, USA) at 37°C incubator for 5 days. Cells were harvested and were subjected to flow cytometry (BD FACS Calibur). Data were analyzed by FlowJo 6.0 software (Tree Star).

### 2.4. Statistical Analysis

SPSS 17.0 software (IBM Corp., Armonk, NY, USA) was used to analyze all data. Data of HCs are shown as mean ± SEM.

## 3. Results

### 3.1. Clinical Characteristics of This GS Patient

**Figure 1:**
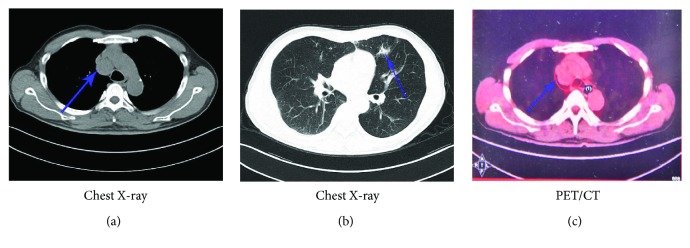
Clinical imaging findings of this GS patient. (a) An anterior mediastinal mass (2.8 cm) in chest X-ray (blue arrow). (b) Signs of pneumonia in chest X-ray (blue arrow) when first hospitalized. (c) Lesion showed via PET/CT (blue arrow).

A 62-year-old man attended a community hospital on April 16, 2012, due to a series of respiration symptoms such as cough and asthma with sputum. Chest X-rays showed a 2.8 cm anterior mediastinal mass and signs of pneumonia (Figures 1(a) and 1(b)). Afterwards, TBNA (transbronchial needle aspiration) failed to collect target tissues after two attempts. So he went to the First Affiliated Hospital of Nanjing Medical University for clear-cutting diagnosis on May 10, 2012. PET/CT scan was subsequently taken, and the SUV value of the lesion was as depicted by 2.4 (Figure 1(c)), which almost excluded the possibility of tumor. Considering his dissipating symptoms of pneumonia, the patient adopted the outpatient follow-up treatment for 7 months until November 27, 2012. In the meantime, the lesions showed no significant regression in computed tomography (CT) imaging during routine follow-ups. On December 4, 2012, the patient was admitted to the Department of Thoracic Surgery for lump resection surgery. The lesion was later pathologically diagnosed with thymoma, B2 type. On February 25, 2013, the patient was readmitted for recurrent respiratory infection. A series of detailed clinical analyses was further carried out, and the results were as follows: fasting blood glucose 7 mmol/l (3.9–6.1 mmol/L), HA1c 7.2% (4.0%–6.4%), positive autoantibodies of glutamic acid decarboxylase (GAD) and insulin autoantibody (IAA), globulin 19.7 g/L (20.0–40.0 g/L), and serum IgG 3.2 g/L (7.0–16.0 g/L), IgM 0.17 g/L (0.4–2.3 g/L), and IgA 0.37 g/L (0.7–4.0 g/L). Complement component 3 (C3) and complement component 4 (C4) results were within reference ranges. The results of routine blood test and blood biochemistry analysis were normal. Serum virus copy number detection including CMV, EBV, HIV, and HBV was negative. But his peripheral lymphocyte subsets were abnormal with low CD4^+^ T cells (16.2%; 30%–40%) and extremely low B cells (0.1%; 9%–14%). He also had slightly decreased muscle tension which improved after surgery. Moreover, this GS patient had mild diarrhea after thymoma resection, which improved without additional treatment. The combination of disrupted peripheral lymphocytes and immunoglobulin and a history of high blood sugar, as well as the diagnosis of thymoma, lead to the patient final diagnosis of GS. Immunoglobulin replacement was immediately and regularly applied to enhance postsurgery immunity supportive therapy until now. Between February 2012 and March 2017, the patient was readmitted several times to our hospital for recurrent respiratory infections due to high susceptibility to gram-negative bacilli or fungi of *Candida albicans* (July 7, 2013; August 19, 2013; May 14, 2015; and March 25, 2016). This GS patient often suffered from cough with sputum, fever of 39°C, and chest pain as well as signs of pneumonia identified via chest CT images. Doctors usually utilized combination therapy including antibiotics (levofloxacin tablets/imipenem/cefodizime) and antifungal (fluconazole) drug to prevent *Candida albicans*, as well as ambroxol for cough with sputum, which eliminated respiratory inflammation and prevented double infection (bacterial-fungal infections). Moreover, the routine monitoring of immunoglobulin, blood sugar, peripheral lymphocyte subsets, and immunoglobulin infusion is still ongoing every 3 months in our GS patient.

### 3.2. Overall Peripheral Immune Cell Distribution in a Postthymectomy GS Patient

**Figure 2:**
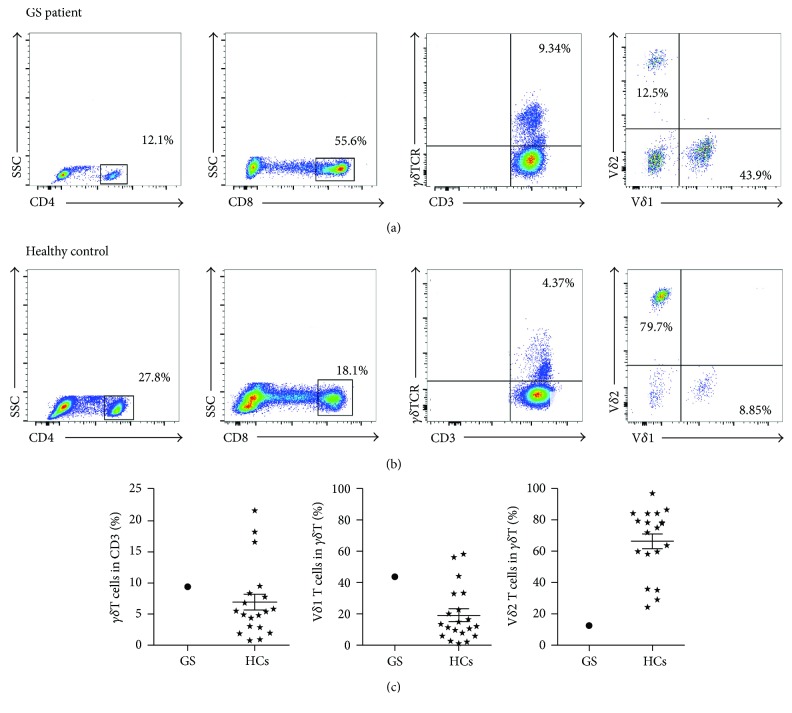
Distribution of peripheral blood immune cells in this GS patient and HCs. Representative of dot pots of CD4^+^ T cell, CD8^+^ T cell, *γδ*T cell, and its subgroups (V*δ*1, V*δ*2) in our GS patient (a) and HCs (b). Detailed statistical graphs of *γδ*T cells and its subgroups in this GS patient and HCs (c; *N* = 20). ⋆: HCs group.

A peripheral blood sample was collected on November 22, 2016 during a routine outpatient examination of a GS patient 4 years after thymectomy. At the time of sample collection, the patient did not show any obvious signs of respiratory infection, but he had a history of recurrent respiratory infections. The proportions of his peripheral immune cells were evaluated by flow cytometry. As shown in Figure 2, this GS patient had an elevated proportion of CD8^+^ T cells (55.6%) and a reduced proportion of CD4^+^ T cells (12.1%) compared with the corresponding levels in healthy control subjects (HCs); furthermore, B cells (1%) were nearly absent in this patient, and he also had an inverted ratio of CD4^+^/CD8^+^ cells. *γδ*T cells play a crucial role in preventing bacterial and fungal infection and act as the first protective barrier of innate immunity. These cells are split into two subgroups based on the classification of the *δ* ligand: V*δ*1 T cells, which are almost located in the epithelial tissue, and V*δ*2 T cells, which account for 70% of the total *γδ*T cells in the peripheral blood. Although the relative proportion of γ*δ*T cells in our GS patient was similar to that in HCs, the percentage of V*δ*2 T cells was particularly low (12.5% versus 66.45 ± 20.79%) in our GS patient. Accordingly, the percentage of V*δ*1 T cells in this patient was higher than that in the HCs (43.9% versus 19.12 ± 17.20%) (Figure 2(c)). This different distribution of *γδ*T cell subsets may be related to the patient's recurrent respiratory infections. It is possible that the altered distribution of peripheral immune cells partly caused an inefficient peripheral immune response.

### 3.3. Evaluation of Treg Cells in Our GS Patient

**Figure 3:**
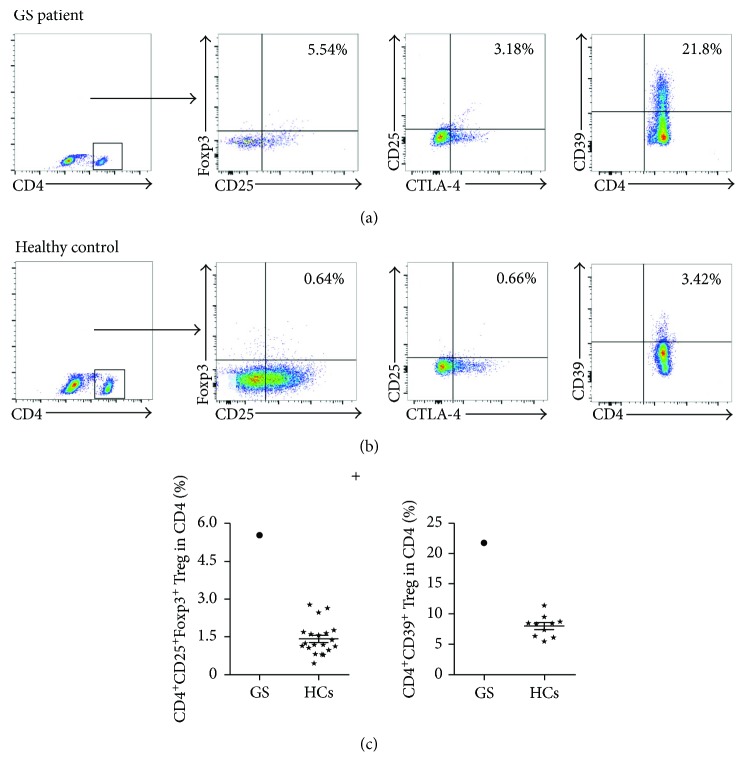
Elevated levels of peripheral CD4^+^ Tregs in this GS patient and HCs. Representing plots of three main markers of CD4^+^ Treg cell subgroups in our GS patient (a) and HCs (b), respectively. The percentages of CD4^+^CD25^+^Foxp3^+^ Tregs (*N* = 20) and CD4^+^CD39^+^ Treg (*N* = 10) in this GS patient and HCs (c). ⋆: HCs group.

**Figure 4:**
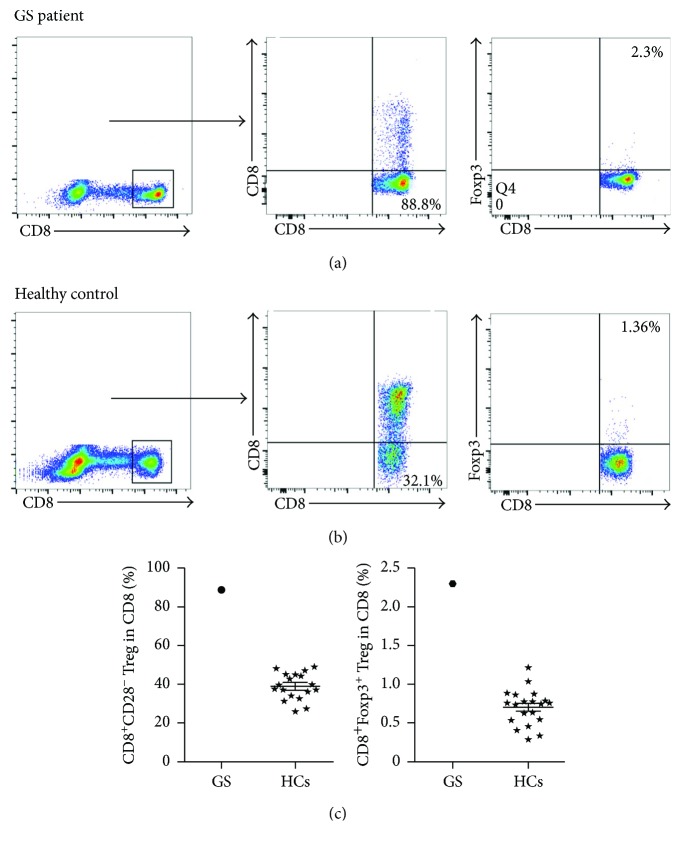
Elevated CD8^+^ Treg infiltration in the peripheral blood of GS patient and HCs. Representative dot plots of peripheral CD8^+^ Treg cells in this GS patient (a) and HCs (b), respectively. Statistical graphs of GS patient and HCs with CD8^+^CD28^−^ Tregs and CD8^+^Foxp3^+^ Tregs (c; *N* = 20, resp.). ⋆: HCs group.

Tregs modulate immune homeostasis that induced immune suppression effect in the peripheral blood. We further analyzed the proportions of five subtypes of Tregs with GS patient via flow cytometry. As shown in Figure 3, the percentages of CD4^+^CD25^+^Foxp3^+^ Treg cells (5.54% versus 1.46 ± 0.71%) and CD4^+^CD25^+^CTLA-4^+^ Treg cells (3.18% versus 0.66%) were higher in our GS patient compared with HCs. Surprisingly, the percentage of CD4^+^CD39^+^ Treg cells was 21.8% in our GS patient, which was much higher than that in the HCs (8.034 ± 1.868%). Recent studies indicated that CD8^+^ Treg cells are increased and associated with tumor stages in human ovarian cancer [6]. As shown in Figure 4, the proportions of CD8^+^CD28^−^ Treg cells and CD8^+^Foxp3^+^ Treg cells in our GS patient were 88.8% (HCs 38.66 ± 6.93%) and 2.3% (HCs 0.71 ± 0.29%), respectively. Additionally, the CD28 expression in our GS patient's immune cells was significantly lower than that in HCs. Thus, the excessive populations of various Treg cells in our GS patient's peripheral blood highlight the severe immune suppression status of this GS patient.

### 3.4. Aberrant Proliferation Capability of CD4^+^ T Cells in Our GS Patient

**Figure 5:**
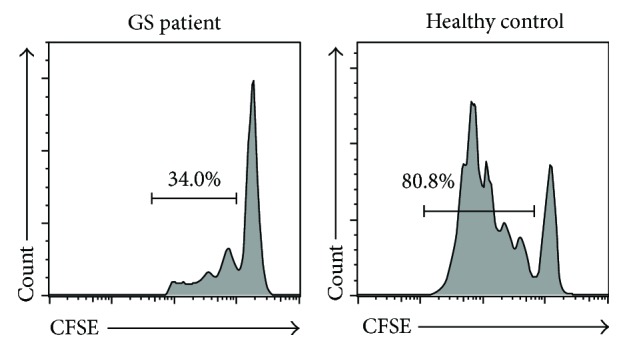
Cell proliferation capacity of peripheral CD4^+^ T cells in this GS patient. Representative dot pots of proliferation assay in this GS patient and HCs.

**Figure 6:**
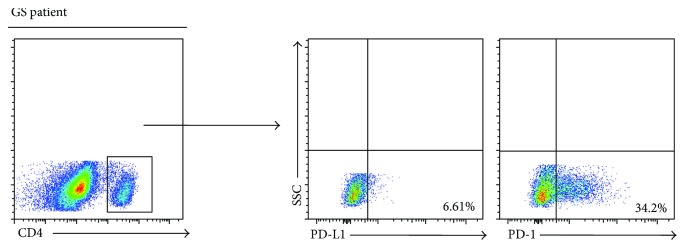
Expression of PD-1 and PD-L1 in this GS patient. Representative dot pots of PD-1 and PD-L1 in this GS patient.

Cell proliferation capacity is an essential index for assessing the presence of an effective immune response. We investigated the ex vivo proliferation of circuiting CD4^+^ T cells from our GS patient via flow cytometry. The result shows that CD4^+^ T cells from our GS patient underwent less proliferation than those from HCs (34.0% versus 80.8%) following stimulation by CD3/CD28; additionally, the cells from the GS patients had lower proliferation rates and fewer divisions (Figure 5). It has been widely reported that immune checkpoints, including programmed death receptor-1 (PD-1) and programmed death ligand-1 (PD-L1), participate in the progression of immune suppression, which also partly correlates with T cell activation and proliferation as well as cytokine secretion. In this study, our GS patient's peripheral CD4^+^ T cells expressed markedly higher levels of PD-1 (34.2% versus 4.2 ± 1.8%) but lower levels of PD-L1 (6.61% versus 10.1 ± 3.9%) than those reported for GS patients (Figure 6); this difference was similar to that reported by previous studies [7]. These results suggest that both poor proliferation and increased PD-1 expression of circulating CD4^+^ T cells, along with a higher level of CTLA-4 expression, may be at least partly associated with the delayed immune reactions to infection in our GS patient.

### 3.5. Cellular Levels of IFN-*γ* and IL-17A in Our GS Patient

**Figure 7:**
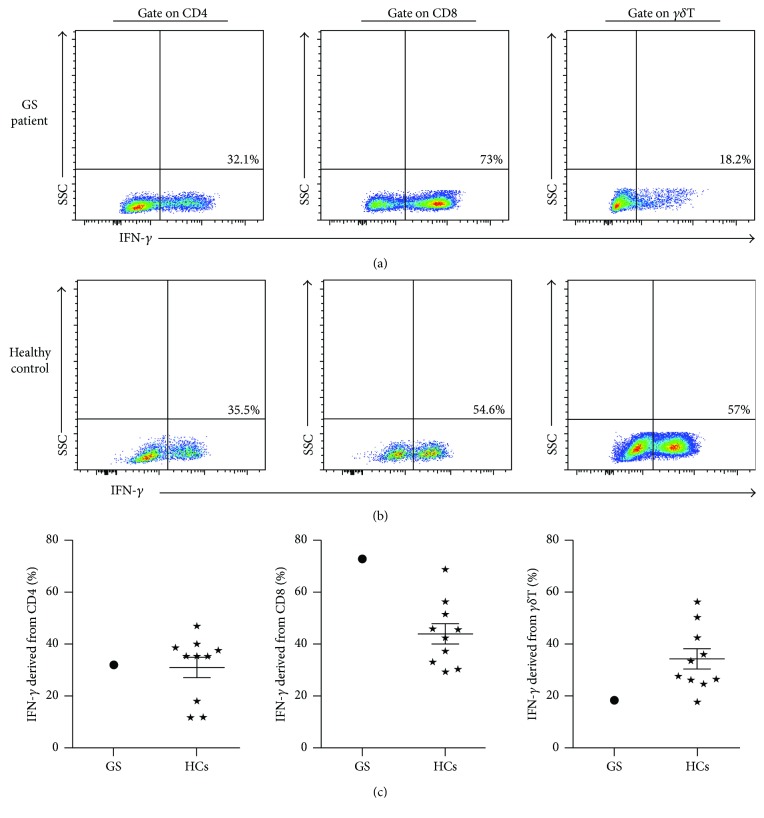
Cellular IFN-*γ* levels originated from the peripheral immune cells in this GS patient and HCs. Representative dot pots of IFN-*γ* derived from CD4^+^ T cells, CD8^+^ T cells, and *γδ*T cells in this GS patient (a) and 10 HCs (b). Detailed statistical graphs of GS patient and HCs with cellular IFN-*γ* (c; *N* = 10, resp.). ⋆: HCs group.

**Figure 8:**
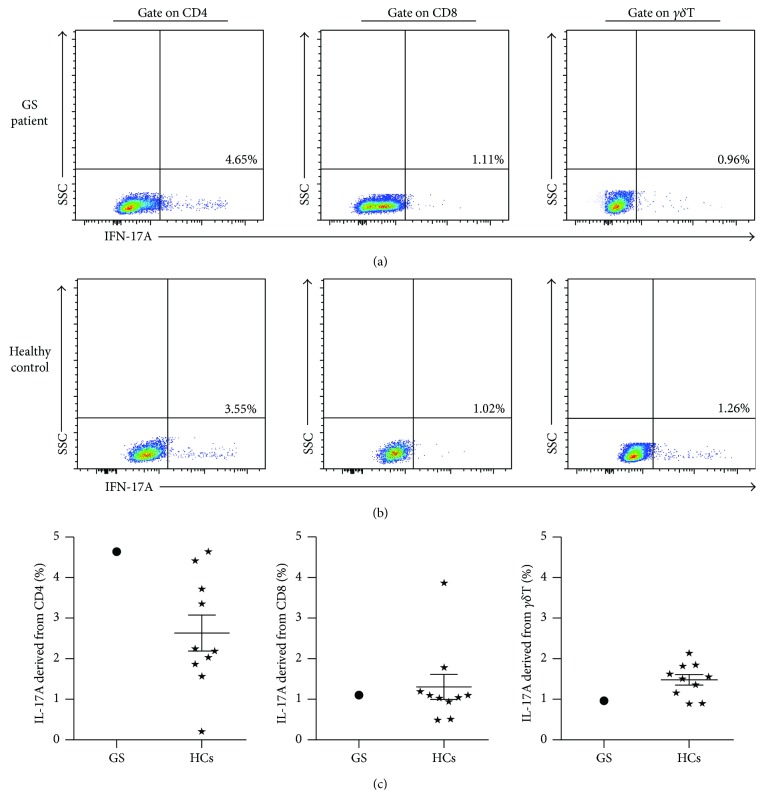
Cellular IL-17A levels derived from circuiting immune cells in this GS patient and HCs. Representative dot pots of IL-17A derived from CD4^+^ T cell, CD8^+^ T cell, and *γδ*T cell in this GS patient (a) and HCs (b). Statistical graphs of intracellular IL-17A in GS patient and HCs (c; *N* = 10, resp.). ⋆: HCs group.

We detected the cellular IFN-*γ* levels produced by ex vivo CD4^+^ T cells, CD8^+^ T cells, and *γδ*T cells from our GS patient, and cells from 10 healthy volunteers were included as controls (Figure 7). The amount of IFN-*γ* produced by CD4^+^ T cells was similar between our GS patient and the HCs (32.5% versus 31.08 ± 12.50%). In contrast, the amount of IFN-*γ* secreted by CD8^+^ T cells was higher in our GS patient than in the HCs (71.1% versus 44.15 ± 12.49%), and the opposite trend was observed in *γδ*T cells (15.1% versus 33.98 ± 12.26%). Additionally, we also found that the circuiting CD4^+^ T cells from our GS patient secreted slightly more IL-17A compared with those from the HCs (4.3% versus 2.62 ± 1.38%) (Figure 8). However, the levels of cellular IL-17A produced by CD8^+^ T cells and *γδ*T cells were not significantly different between our GS patient and the HCs (1.20% versus 1.37 ± 0.91%; 2.70% versus 1.42 ± 0.41%, resp.). These results illustrate the abnormality of cellular cytokine levels in our GS patient, which may be linked to the recurrent respiratory infections suffered by this patient.

## 4. Discussion

The thymus is a vital organ responsible for the differentiation and maturation of the immune system in early childhood and adolescence. GS is a rare type of thymoma and has high susceptibility to encapsulated bacteria in the respiratory system, as well as opportunistic viral and fungal invasions, due to serious combined T and B cell immunodeficiency [8, 9]. GS patients often display CD4^+^ T cell lymphopenia and an inverse ratio of CD4^+^/CD8^+^ [10]. The present case also showed this abnormality of circuiting lymphocytes, with lower than normal percentages of CD4^+^ T cells and a reversed ratio of CD4^+^/CD8^+^ cells. Furthermore, we found that the patient's peripheral CD4^+^ T cells had a lower proliferation capacity and a higher expression level of PD-1. Further experiments are needed to determine if the altered PD-1 levels in our GS patient contribute to the reduced proliferation of the patient's CD4^+^ T cells. In addition, the higher expression of CTLA-4 in the CD4^+^ T cells (Figure 3) of our GS patient may function to capture and remove CD80 and CD86; if so, these proteins would be unable to trigger CD28, further impairing T cell activation [11]. The altered peripheral immune milieu in our GS patient may at least partially explain his repeated respiratory infections.

*γδ*T cells participate vigorously in the first line of innate immunity [12, 13]. However, their functions in GS have not been fully elucidated yet. Here, although the percentage of peripheral *γδ*T cells in our GS patient was similar to that of HCs, the amount of V*δ*2 T cells was extremely low, resulting in a relatively higher proportion of V*δ*1 T cells in the peripheral blood. Previous studies have revealed that peripheral V*δ*1 T cells exert strong anti-infection and antitumor effects through binding to MHC class I chain-related gene A/B (MICA/B) or UL16-binding proteins (ULBPs), and these cells display a strong cytotoxic ability upon stimulation with PHA (polyhydroxyalkanoates) and IL-7 *in vitro* [14–18]. Furthermore, upregulated levels of peripheral V*δ*1 T cells followed by an inverse ratio of V*δ*2/V*δ*1 T cells were reported to participate in antiviral immunity during HIV infection [19]. However, the increased proportion of V*δ*1 T cells in this GS patient did not seem to exert an effective function, given the repeated respiratory infections suffered by the patient. The role of these cells in GS is in need of further exploration.

There are no previous reports on the cellular expression of IL-17A in the peripheral blood of GS patients; however, since this cytokine acts as a culprit in the aggressive progressions of various chronic inflammatory diseases [20], we investigated its levels in our GS patient. We separately measured the IL-17A produced by CD4^+^ T cells, CD8^+^ T cells, and *γδ*T cells but did not detect any obvious differences between the cells from our GS patient and those from HCs. However, tissue-derived IL-17A, which can recruit myeloid-derived suppressor cells (MDSCs), was reported to energetically participate in the immunosuppression milieu in human colorectal cancer [21]. Therefore, future work should investigate the significance of IL-17A that has infiltrated into the lesions of our GS patient.

Peripheral V*δ*2 T cells can secrete IFN-*γ* [15], but the level of IFN-*γ* secreted by *γδ*T cells from our GS patient was significantly lower compared with HCs, and this difference may be involved with the observed mild alternations in the CD4^+^ T cell-derived IFN-*γ* levels in this patient (Figure 7). Because IFN-*γ* production by CD8^+^ T cells may partially compensate for insufficient cellular IFN-*γ* [22], we also measured the expression levels of IFN-*γ* produced by these cells. Data from numerous cytokine profiles suggest that the recurrent respiratory infections suffered by our GS patient could be related to the intracellular expressions of IL-17A and IFN-*γ*. However, functional evaluations of immune elements, especially the conventional cytotoxic effect of IFN-*γ* expressed by CD8^+^ T cells need to be performed in future work. Furthermore, the complete cytokine profiles of GS patients should be measured as part of a comprehensive assessment of GS patients.

Treg cells play an essential role in preserving self-immune tolerance through the effective suppression of self-recognizing immune cells. Multifunctional circulating Treg cells with lower Foxp3 expression have been reported in immunodeficient patients [23–28]. The levels of peripheral CD4^+^CD25^+^Foxp3^+^, CD4^+^CD25^+^CTLA-4^+^, CD4^+^CD39^+^, CD8^+^CD28^−^, and CD8^+^Foxp3^+^ Treg cells were all relatively higher in our GS patient, which indicate that the patient's peripheral blood had an immunosuppressed milieu. One study reported that CD28-deficient mice exhibit weakened T cell proliferation potential after TCR stimulation and a passive B cell response, despite retaining normal cytotoxic effects [29]. Therefore, it is possible that the lower expression of CD28 in our GS patient may have been partially responsible for the observed defects in his immune responses, particularly after the patient's thymus removal, which can eliminate the immature lymphocytes.

Notably, our GS patient had a higher level of CD4^+^CD39^+^ Treg cell infiltration (Figure 3). CD39 is an enzyme that hydrolyzes adenosine triphosphate (ATP) to adenosine diphosphate (ADP) and adenosine monophosphate (AMP) with the assistance of CD73. However, the accumulation of adenosine is considered another metabolism-related mechanism of the immunosuppressive effect caused by alterations in Treg cell populations [30]. Given that our GS patient repeatedly suffered from respiratory infections, we plan to conduct a follow-up study detecting the levels of circulating and lesion-based adenosine and evaluating the potential correlation between adenosine levels and Treg cells in GS progression.

Some GS patients lack peripheral B cells, have stalled pre-B cells, and/or exhibit weakened maturation of erythroid and myeloid precursors due to bone marrow defects [31]. Interestingly, this abnormal lymphocyte distribution is not constrained to only within the bone marrow but is also observed in the lymph nodes, lymphoid tissue, and spleen [9]. Unfortunately, it is impossible to exclude the effects of the surgery itself and the postsurgery immune replacement therapy on the results of our study because there is inadequate preoperative and postoperative information available to evaluate their effect on our laboratory data. Thus, we will continue to evaluate the peripheral immune status of this GS patient during follow-up visits over the coming year, and we will collect a broader set of data on any additional GS patients we encounter.

## 5. Conclusions

In summary, we determined the peripheral immune cell distribution and corresponding intracellular cytokine secretion levels of a GS patient. Although there are still no effective protocols for treating GS, except for immunoglobulin replacement, our work expanded knowledge of the postthymectomy immunosuppressed status in this condition, which might provide potential targets for successive supportive treatment.

## Supplementary Materials

Supplementary Materials Figure S1(A): the statistical comparison of IFN-*γ* ^+^ IL-17A^+^ cells in CD4^+^ T cells with this GS patient and HCs (*N* = 10). (B): representative dot plot of IFN-*γ* ^+^ IL-17A^+^ cells derived from peripheral CD4^+^. T cells in this GS patient and HCs.

## References

[1] Good R. A. (1954). Absence of plasma cells from bone marrow and lymph nodes following antigenic stimulation in patients with a gamma globulinemia. *Revue d'Hématologie*.

[2] Dong J. P., Gao W., Teng G. G., Tian Y., Wang H. H. (2017). Characteristics of Good’s syndrome in China: a systematic review. *Chinese Medical Journal*.

[3] Kelleher P., Misbah S. A. (2003). What is Good’s syndrome? Immunological abnormalities in patients with thymoma. *Journal of Clinical Pathology*.

[4] Ohuchi M., Inoue S., Hanaoka J. (2007). Good syndrome coexisting with leukopenia. *The Annals of Thoracic Surgery*.

[5] Speiser D. E., Ho P. C., Verdeil G. (2016). Regulatory circuits of T cell function in cancer. *Nature Reviews Immunology*.

[6] Wu M., Chen X., Lou J. (2017). Changes in regulatory T cells in patients with ovarian cancer undergoing surgery: preliminary results. *International Immunopharmacology*.

[7] Campbell D. E., Tustin N. B., Riedel E. (2009). Cryopreservation decreases receptor PD-1 and ligand PD-L1 coinhibitory expression on peripheral blood mononuclear cell-derived T cells and monocytes. *Clinical and Vaccine Immunology*.

[8] Tarr P. E., Sneller M. C., Mechanic L. J. (2001). Infections in patients with immunodeficiency with thymoma (Good syndrome). Report of 5 cases and review of the literature. *Medicine*.

[9] Miller J. F. A. P. (2002). The discovery of thymus function and of thymus-derived lymphocytes. *Immunological Reviews*.

[10] Kelesidis T., Yang O. (2010). Good’s syndrome remains a mystery after 55 years: A systematic review of the scientific evidence. *Clinical Immunology*.

[11] Ahmed A., Mukherjee S., Nandi D. (2009). Intracellular concentrations of Ca^2+^ modulate the strength of signal and alter the outcomes of cytotoxic T-lymphocyte antigen-4 (CD152)-CD80/CD86 interactions in CD4^+^ T lymphocytes. *Immunology*.

[12] Paul S., Shilpi, Lal G. (2015). Role of gamma-delta (*γδ*) T cells in autoimmunity. *Journal of Leukocyte Biology*.

[13] Zheng R., Yang Q. (2014). The role of the *γδ* T cell in allergic diseases. *Journal of Immunology Research*.

[14] Wu D., Wu P., Wu X. (2015). *Ex vivo* expanded human circulating V*δ*1 *γδ*T cells exhibit favorable therapeutic potential for colon cancer. *OncoImmunology*.

[15] Corvaisier M., Moreau-Aubry A., Diez E. (2005). V*γ*9V*δ*2 T cell response to colon carcinoma cells. *Journal of Immunology*.

[16] Wu J., Groh V., Spies T. (2002). T cell antigen receptor engagement and specificity in the recognition of stress-inducible MHC class I-related chains by human epithelial gamma delta T cells. *Journal of Immunology*.

[17] Eleme K., Taner S. B., Önfelt B. (2004). Cell surface organization of stress-inducible proteins ULBP and MICA that stimulate human NK cells and T cells via NKG2D. *The Journal of Experimental Medicine*.

[18] Lu J., Aggarwal R., Kanji S. (2011). Human ovarian tumor cells escape *γδ* T cell recognition partly by down regulating surface expression of MICA and limiting cell cycle related molecules. *PLoS One*.

[19] Paoli P., Gennari D., Martelli P. (1991). A subset of *γδ* lymphocytes is increased during HIV-1 infection. *Clinical & Experimental Immunology*.

[20] Mumm J. B., Oft M. (2013). Pegylated IL-10 induces cancer immunity: the surprising role of IL-10 as a potent inducer of IFN-*γ*-mediated CD8^+^ T cell cytotoxicity. *BioEssays*.

[21] Wu P., Wu D., Ni C. (2014). *γδ*T17 cells promote the accumulation and expansion of myeloid-derived suppressor cells in human colorectal cancer. *Immunity*.

[22] Thongprayoon C., Tantrachoti P., Phatharacharukul P., Buranapraditkun S., Klaewsongkram J. (2013). Associated immunological disorders and cellular immune dysfunction in thymoma: a study of 87 cases from Thailand. *Archivum Immunologiae et Therapiae Experimentalis*.

[23] Azizi G., Hafezi N., Mohammadi H. (2017). Abnormality of regulatory T cells in common variable immunodeficiency. *Cellular Immunology*.

[24] Carter C. R. D., Aravind G., Smalle N. L., Cole J. Y., Savic S., Wood P. M. D. (2013). CVID patients with autoimmunity have elevated T cell expression of granzyme B and HLA-DR and reduced levels of Treg cells. *Journal of Clinical Pathology*.

[25] Yu G. P., Chiang D., Song S. J. (2009). Regulatory T cell dysfunction in subjects with common variable immunodeficiency complicated by autoimmune disease. *Clinical Immunology*.

[26] Arumugakani G., Wood P. M. D., Carter C. R. D. (2010). Frequency of Treg cells is reduced in CVID patients with autoimmunity and splenomegaly and is associated with expanded CD21lo B lymphocytes. *Journal of Clinical Immunology*.

[27] Melo K. M., Carvalho K. I., Bruno F. R. (2009). A decreased frequency of regulatory T cells in patients with common variable immunodeficiency. *PLoS One*.

[28] Arandi N., Mirshafiey A., Jeddi-Tehrani M. (2013). Evaluation of CD4^+^CD25^+^FOXP3^+^ regulatory T cells function in patients with common variable immunodeficiency. *Cellular Immunology*.

[29] Krawczyk P., Adamczyk-Korbel M., Kieszko R., Korobowicz E., Milanowski J. (2007). Immunological system status and the appearance of respiratory system disturbances in thymectomized patients. *Archivum Immunologiae et Therapiae Experimentalis*.

[30] Hu G., Wu P., Cheng P. (2017). Tumor-infiltrating CD39+ γδTregs are novel immunosuppressive T cells in human colorectal cancer. *OncoImmunology*.

[31] Tarr P. E., Lucey D. R., for the Infectious Complications of Immunodeficiency with Thymoma (ICIT) Investigators (2001). Good’s syndrome: the association of thymoma with immunodeficiency. *Clinical Infectious Diseases*.

