# Supplementary material for: Aberrant Peripheral Immune Function in a Good Syndrome Patient
Source: J Immunol Res. 2018 Apr 23;2018:6212410. doi: 10.1155/2018/6212410 (PMC5937423; doi:10.1155/2018/6212410)
Supplement: Supplementary Materials — Figure S1(A): the statistical comparison of IFN-γ + IL-17A+ cells in CD4+ T cells with this GS patient and HCs (N = 10). (B): representative dot plot of IFN-γ + IL-17A+ cells derived from peripheral CD4+. T cells in this GS patient and HCs. [file 6212410.f1.pdf]

Supplemental Figure S1

Fig.S1

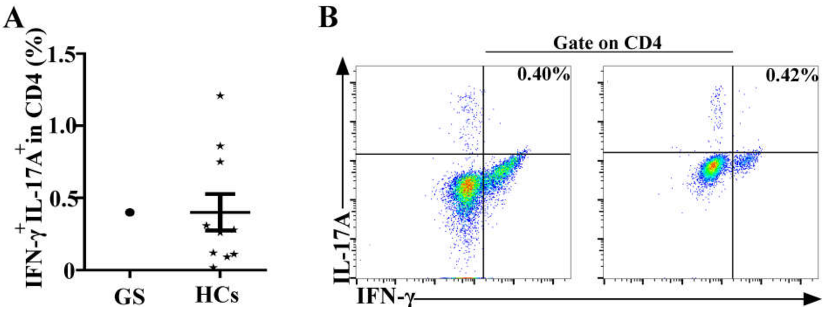

Figure S1.

A. **The statistical comparison of IFN-γ^{+} IL-17A^{+} cells in CD4^{+} T cells with this GS patient and HCs (N=10).**

B. **Representative dot plot of IFN-γ^{+} IL-17A^{+} cells derived from peripheral CD4^{+} T cells in this GS patient and HCs.**
